# The Operative Treatment of Genu-Valgum

**Published:** 1877-10

**Authors:** 


### Selections.

---

THE OPERATIVE TREATMENT OF GENU-VALGUM.—A boy of 18, presenting a bad case of knock-knee of twelve years standing, was admitted into the Aberdeen Infirmary on the 9th of April, 1876. The patient was a robust, strongly muscular lad, well nourished, but, owing to the deformity, short of stature. The deformity was so great, that, when the patient stood erect, the hand could "be passed between the transposed knees." Mechanical treatment was used for some time, until it became evident that it was useless to pursue it further, and operative measures were then decided upon. On May 17th, 1876, the patient was chloroformed, and the right knee was flexed and the thigh turned outward. A long and strong tenotomy knife (Adams's) was introduced through the skin, 3½ inches above the tip of the internal condyle on the inner side of the thigh, and so far back as to be opposite the ridge of bone running between the linea aspera and the condyle. The blade was then carried "forward, downward and outward," over the front of the femur. When the point of the blade could be felt under the skin, in the groove between the condyles (the normal position of the patella in the flexed position), the cutting edge was pressed against the bone, and the soft parts and periosteum divided in withdrawing the knife. The external wound thus made was one-third of an inch long, and terminated in the cavity of the joint. Adams's saw for subcutaneous division of the neck of the femur was then introduced, and as soon as it was estimated that the condyle was entirely separated, and that the saw was approximating the poplitcal space, it was withdrawn. The knee was now completely extended, and the limb was forcibly straightened laterally; the hands of the operator forming the power, and his knee the fulcrum, by which this part of the operation was accomplished. The undivided portion of the condyle gave way with a snap upon the application of a moderate amount of force, and the limb immediately became straight. The limb was then simply bandaged to a long Liston splint, and the patient placed in the recumbent position. The other limb was similarly operated upon, on June 6th. Lister's antiseptic measures were carefully carried out, and the reaction in each case was almost *nil.* After the first operation the temperature never rose above 99.8 deg., in the second reaching 100 deg. only. The apparatus was removed after the first operation on the sixteenth day, and passive motion resorted to. Fifteen days after the second operation, passive movements were also instituted. There was never any pain, and five weeks after the last operation, the patient was allowed to walk. The movements of the limbs became perfectly normal (the detached condyles uniting perfectly in their acquired positions); and on July 21st, 1876, the patient was discharged, "walking perfectly."—*Edinburgh Medical Journal.*

